# Mothers’ Use of Social Media to Inform Their Practices for Pumping and Providing Pumped Human Milk to Their Infants

**DOI:** 10.3390/children3040022

**Published:** 2016-10-31

**Authors:** Rei Yamada, Kathleen M. Rasmussen, Julia P. Felice

**Affiliations:** Division of Nutritional Sciences, Cornell University, Ithaca, NY 14853, USA; ry84@cornell.edu (R.Y.); kathleen.rasmussen@cornell.edu (K.M.R.)

**Keywords:** breastfeeding, human milk expression, expressed human milk feeding

## Abstract

Despite U.S. mothers’ wide adoption of pumps and bottles to provide human milk (HM) to their infants, mothers lack comprehensive, evidence-based guidelines for these practices. Thus, some women use online sources to seek information from each other. We aimed to characterize the information women sought online about pumping. We used data provided by ~25,000 women in an open cohort within a discussion forum about parenting. We examined 543 posts containing questions about providing pumped HM cross-sectionally and longitudinally in three time intervals: prenatal, 0 through 1.5 months postpartum, and 1.5 to 4.5 months postpartum. We used thematic analysis with Atlas.ti to analyze the content of posts. During pregnancy, women commonly asked questions about how and where to obtain pumps, both out-of-pocket and through insurance policies. Between 0–1.5 months postpartum, many mothers asked about how to handle pumped HM to ensure its safety as fed. Between 1.5–4.5 months postpartum, mothers sought strategies to overcome constraints to pumping both at home and at work and also asked about stopping pumping and providing their milk. Women’s questions related to ensuring the safety of pumped HM represent information women need from health professionals, while their questions related to obtaining pumps suggest that women may benefit from clearer guidelines from their insurance providers. The difficulties women face at home and at work identify avenues through which families and employers can support women to meet their goals for providing HM.

## 1. Introduction

In the United States, mothers whose infants are fed human milk (HM) have rapidly and widely adopted using pumps and bottles to provide HM in place of some or all feeding at the breast [1]. Of the mothers who provided HM to their infants in a recent longitudinal survey cohort, the Infant Feeding Practices Study II (IFPS II) [1], 92% pumped at some point, 25% pumped regularly between 1.5–4.5 months, and more than 50% began pumping in their first week postpartum. Pumped HM is nationally endorsed as equivalent to HM provided directly at the breast [2], but very few data compare providing pumped HM or pumping to feeding at the breast for outcomes to infants or mothers. Thus, mothers who pump and provide pumped HM do not benefit from comprehensive evidence-based guidelines for these practices. This is troubling in light of recent studies, that suggest that pumping or providing pumped HM may not yield the same benefits to infants and mothers as feeding at the breast and may incur additional risks for infants [3,4,5,6,7,8,9,10,11,12] and burdens for mothers [13].

Because of mothers’ need or desire to work outside the home in their first postpartum year [14,15,16,17,18], and the many work-related obstacles to feeding at the breast [19], working mothers are more likely to pump regularly in their first postpartum year than non-working mothers [19]. Previous qualitative work, which includes in-depth, semi-structured interviews with lactating mothers [13], suggests that financial and work-related constraints to pumping may have an important role in mothers’ potential for success with pumping their milk. It has also been shown that pumping and providing pumped HM requires a range of tasks and considerations that are not necessary with feeding at the breast, such as those related to collecting and storing pumped HM at work and at home.

The impetus for this work is that, in the absence of comprehensive, evidence-based guidelines for pumping and providing pumped HM, and without the benefit of generational knowledge and influence that may be available with feeding at the breast [20], women must seek help themselves. Women in previous qualitative samples commonly reported finding information using the Internet [13], including social media, such as discussion forums that facilitate information sharing among peers. Recent data suggest that information shared on social media influences personal health practices [21]. Thus, we expected that online discussion forums might provide valuable insight into the information women seek about pumping and providing pumped HM.

We aimed to characterize the information women sought over time about pumping on an online parenting discussion forum. The information women seek from each other may represent information that is not currently accessible to them from professional resources, and understanding their questions will provide knowledge of the information needed to better support women in their practices of pumping and providing HM.

## 2. Materials and Methods

We conducted a qualitative, longitudinal and cross-sectional analysis of data provided by a cohort of women within an online discussion forum whose infants were born in April 2014. We used BabyCenter [22], one of the largest online forums related to pregnancy and parenting in the first year, and monitored the group of women from one month before delivery to approximately 4.5 months postpartum.

### 2.1. Longitudinal Design

To investigate how the number and nature of questions changed over time, we examined questions women asked about pumping for three age groups: when women were pregnant, from delivery through 1.5 months postpartum, and from 1.5 to 4.5 months postpartum. These infant age groups were chosen to reflect prior analyses of the IFPS II by others [23], who divided mothers into three mutually exclusive groups (1.5–4.5, 4.5–6.5, and 6.5–9.5 months postpartum). We focused on questions posted up until 4.5 months and included 1 month before delivery based on the observations of previous work, which showed that women illustrated the importance of the early postpartum period to establishing and maintaining their practices for providing HM [13].

### 2.2. Cross-Sectional Design

Previous qualitative work [13] has identified five important themes to investigate: (1) choosing and purchasing pumps; (2) storing and preparing pumped HM; (3) strategies for and difficulties with pumping; (4) integrating pumping into work; and (5) stopping pumping. Based on previous studies, we expected some of the women’s questions about pumps, pumping, and related practices to be directly linked to their perceptions of and intentions for their infants to be fed pumped HM. For example, reports from mothers in previous samples suggest that their questions about HM storage practices relate to their desire to provide safe HM. Thus, the practice of preparing HM was included into the second theme of storing HM. However, we did not explicitly search for bottle-feeding HM, as that would have substantially widened the scope of this work and increased the amount of included data. Some predetermined topics within each main theme were identified from the literature [1] and previous qualitative work [13] to facilitate the analysis of data.

### 2.3. Study Sample

The unit of analysis was publicly accessible posts on BabyCenter forums [22] that included questions related to pumping, as described above. Posts were drawn from the April 2014 Birth Club on BabyCenter, a self-selected cohort of women whose babies were born in that month and whose demographic characteristics were otherwise unidentified. We chose BabyCenter because it has a search engine that facilitates research by enabling the retrieval of data using keywords. Each Birth Club on BabyCenter has approximately 25,000 members, with an open group feature that permits women to join or leave the group at any time.

### 2.4. Data Collection and Cleaning

We identified posts to be examined for inclusion in analyses using relevant keywords such as “pump”, “pumping”, and “expressing”. We saved all posts about pumping from the web as PDF files. Posts were divided in the three infant age groups, according to the date of the post (i.e., 1–31 March 2014 for the prenatal interval, 1 April 2014–15 May 2014 for the 0–1.5 months postpartum interval, and 16 May 2014–15 August 2014 for the 1.5–4.5 months postpartum interval). Inasmuch as the majority of the women did not include their infants’ due dates or ages in their posts, we assumed a mean sample due date of 15 April 2014. We then used 1 April 2014 as a reference point for infants’ birth dates in this sample, as the median length of pregnancy in the U.S. is about two weeks short of full term [24]. Thus, the data collection period was from 1 March 2014–15 August 2014. Posts were subsequently categorized into five themes by the topic of the question. The sample of posts was publicly accessible, did not include any identifying information, and was not considered to be human subjects research by the Cornell University Institutional Research Board, thereby excluding the need for IRB approval or participant consent.

**Figure 1:**
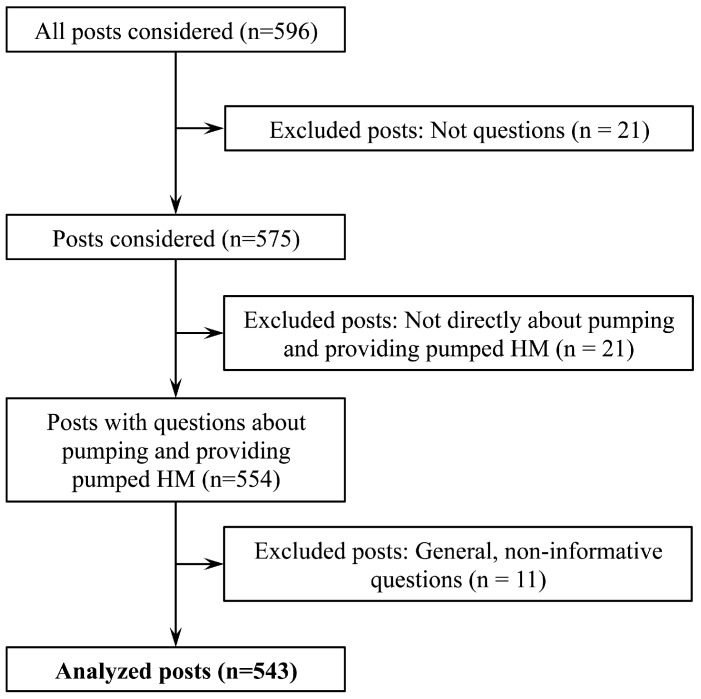
Inclusion flowchart of data for analyses. This figure shows the process to determine which posts, among the 596 posts screened, were included into the final analyses.

Throughout the sorting process, some posts were excluded from analyses. We excluded posts that did not include questions (n = 21), such as those that were sharing information about pumping but not as a question. We also excluded posts in which the question was not about pumping or providing pumped HM (n = 21), and general, non-informative questions about pumping or providing pumped HM (n = 11). Non-informative posts included questions and polls that asked about others’ general opinions on pumping and providing pumped HM instead of a specific aspect of these practices. The inclusion of data into analyses is described in Figure 1.

### 2.5. Data Analysis

Included posts were examined with a thematic analysis using open- and closed-coding in Atlas.ti (Version 7.0; Scientific Software Development GmbH, Berlin, Germany). Primary analysis was done by a single coder (RY), using the code book created by JPF for prior qualitative work [13], and modified by JPF and RY, both for prior work, and again for the analyses reported here. Coding was ongoing, iterative, and discussed weekly by JPF and RY to reflect emerging topics and to verify accuracy. Posts were analyzed by each of the five main themes, and then by time period, from earliest to latest. Because the number of weeks in each infant age interval differed, we compared the number of posts per week by dividing the number of posts in each interval by the number of weeks in that interval—i.e., by four weeks for prenatal posts, six weeks for posts between 0–1.5 months postpartum, and 13 weeks for posts between 1.5–4.5 months postpartum. Quotes presented here are reported by an infant age interval—or, when available, an infant’s age—at the time of data collection. Presented quotes were selected for clarity in illustrating the topic being described and for efficiency in illustrating more than one topic or idea (e.g., a question in addition to an attitude, perception, or practice related to that question).

## 3. Results

The April 2014 Birth Club had 18,303 members at the beginning of the data collection period and steadily grew through 4.5 months postpartum. Women in the April 2014 Birth Club posted 543 eligible questions, which were grouped by theme and infant age group (Table 1). Women asked the most questions per week between 0–1.5 months postpartum, fewer questions between 1.5–4.5 months postpartum, and the fewest in the month before they delivered their infants. The frequency of posts differed across the five main themes, with most posts related to strategies for and difficulties with pumping.

Women’s questions related to strategies for and difficulties with pumping were similar, whether they were asking about pumping at home, away from home, or both. As a result, findings from this theme, and those related to integrating pumping into work, were grouped together. Examples of topics raised by women whose posts were analyzed are listed in Table 2.

### 3.1. Choosing and Purchasing Pumps

#### 3.1.1. Longitudinal Results

Women commonly asked questions about choosing pumps in the prenatal period, with some describing a desire to obtain their pumps before they delivered their infants or concerns that their insurance policies would not provide pumps until after they delivered. Before they delivered their infants, women also asked about how long it would take to obtain a pump through their insurance policies, and, thus, when they should start the process so as to receive a pump by the time they planned to use it.

#### 3.1.2. Cross-Sectional Results

Questions about obtaining pumps indicated that women commonly purchased pumps out-of-pocket or through their insurance policies. Women who asked about purchasing a pump out-of-pocket either did not have an insurance policy that covered a pump or were unsatisfied with the pumps covered by their insurance policies. Some of the women who planned to purchase a pump out-of-pocket asked about how and where to obtain an affordable pump.

I really want to get a breast pump but there just so expensive i’ve looked online and my [husband] is just like no thats to much but ive really want to get one until i seen the prices! Any one know of a good cheaper place to get them? Cus 200+ is just to much!

—prenatal

Some women whose insurance policies covered the purchase of a pump asked about which brands and models were offered by their policies, and about the processes by which they could obtain a pump through their policy. For example, some asked whether a doctor’s prescription was needed and whether and how they should obtain a pump from a special vendor.

[Insurance provider] told me insurance would cover a breast pump. Well fast forward & I’m just a couple of days away from delivery & need to get mine ASAP. Has anyone with the same plan been able to pick up the pump from a med supply store? Are they shipped only? And did you need to get a prescription first?

—prenatal

### 3.2. Storing and Preparing Pumped HM

#### 3.2.1. Longitudinal Results

Most questions about storing and preparing pumped HM were posted after mothers delivered their infants. Questions they asked in the prenatal period related to selecting supplies, such as bags and bottles to store and feed HM. In some of these questions, women described considerations, such as the maintenance required to keep supplies clean. In contrast, questions they asked after delivery commonly related to storing and preparing pumped HM that was safe for their infants (i.e., not spoiled or contaminated).

#### 3.2.2. Cross-Sectional Results

Mothers sought information about how to safely store their pumped HM, such as where, how, and in what containers to store their milk until it was fed. For example, they asked about how long pumped HM could be kept at room temperature or stored in the refrigerator or freezer: “How long do you store in the fridge? 5 days is the max? Then do you freeze the milk in bags?” (0–1.5 months postpartum). Some mothers asked whether it was safe for their infants to be fed milk that was stored and/or prepared by combining HM from different pumping sessions: “I am pumping after I feed and only getting about an ounce or less and would like to get to at least 4 if im freezing it. Can i combine??” (0–1.5 months postpartum).

Mothers also sought information about how to safely prepare their pumped HM to be fed. They asked if infants should be fed fresh milk instead of frozen milk to ensure the safety of the milk, while describing that they did not want to waste their stock of frozen milk by providing only fresh milk. Mothers also asked whether bottled HM that had been refused or unfinished by their infants was safe to be fed to their infants later: “Is it safe once he starts drinking out of one bottle and he doesn’t finish all of it to save the rest in the fridge and give it to him at the next feeding?” (0–1.5 months postpartum). Further, mothers asked about the effect of consuming alcohol, caffeine, or medications on the safety of their milk. Some asked if they should pump and discard after consumption, and if so, for how long to do it to ensure the safety of the pumped HM as fed.

Some questions focused on whether or not and how mothers could recognize the safety or nutritional value of their pumped HM by its taste, smell, and appearance. For example, some mothers asked how to interpret changes to the smell or taste of their pumped HM after refrigeration and freezing.

I usually store the milk in the fridge for a couple of days and then freeze it if its not consumed…I noticed/tasted milk freshly expressed – it has a sweet taste. Milk stored in fridge, then in the freezer, when unfrozen, it seems slight acidic. Is this normal? [sic]

—postpartum (1.5–4.5 months)

Other mothers asked how to interpret the appearance of their pumped HM: “[My breast milk] has the thinnest layer of creamy hind milk on top after it’s been in the fridge for a while and separated. What’s up with that? I think it should be fattier,” (1.5–4.5 months postpartum).

### 3.3. Strategies for and Difficulties with Pumping and Integrating Pumping into Work

#### 3.3.1. Longitudinal Results

Mothers posted most questions about strategies for and difficulties with pumping between 0–1.5 months postpartum, when they were first attempting to pump. However, mothers asked most questions about pumping at work outside the home between 1.5–4.5 months postpartum, when they were preparing to, or had already, returned to work.

#### 3.3.2. Cross-Sectional Results 

Many questions related to strategies for and difficulties with pumping came from mothers who were transitioning from primarily feeding at the breast to pumping regularly. Mothers commonly indicated that this transition was in response to or in preparation for returning to work. Some mothers asked for advice about overcoming physical difficulties, such as nipple pain or bleeding from using pumps or engorgement from not pumping enough. Some mothers had already unsuccessfully tried other strategies for these problems.

It seems like everyday my breasts get fuller, and never empty…I’ve tried heating pads, but only helped for a few pumps. Today my breasts are so sore, my nipples are cracked even though I use lanolin before and after pumping. Any suggestions?

—postpartum (0–1.5 months)

Other mothers had questions about or difficulties with their pumps, and sought information about using pump features and fixing pump malfunctions.

Some of the mothers’ questions related to the barriers to pumping that they either experienced or anticipated. These barriers included the presence of infants who needed care, workloads that were heavy or inflexible, a lack of designated place to pump, and travel: “How do you handle meetings (both in and out office) and pumping? I’m more worried about a few all day meetings I have off-site. Do you bring your pump with you?” (1.5–4.5 months postpartum).

### 3.4. Stopping Pumping

#### 3.4.1. Longitudinal Results

Mothers only posted questions about stopping pumping in the postpartum period, and most often between 1.5–4.5 months postpartum.

#### 3.4.2. Cross-Sectional Results

In their questions about stopping pumping, mothers described a number of considerations, including their infants’ age, their own emotional and physical burdens related to pumping, and how important it was to them to provide HM.

Some mothers described reasons for stopping pumping, such as feeling burdened from the additional tasks of pumping, and asked questions related to whether those reasons were appropriate or acceptable. Some mothers were generally interested in what others were doing or planned to do, or asked for advice on when they should plan to stop pumping.

When will you stop pumping? …I guess I’m not sure what my end goal should be. A certain age? A certain amount frozen? What is your situation, how long will you pump for? Maybe hearing others situations will help me decide what I want to do.

—postpartum (0–1.5 months)

Some posts about stopping pumping were related to the mothers’ personal experiences with stopping or transitioning away from pumping. Some mothers asked general questions about this experience, while others asked questions about specific challenges they anticipated related to stopping pumping, such as guilt.

I have been exclusively pumping since day 1 and [my baby] is now 16 weeks old. For various reasons I am ready to give up on pumping and put him on formula. However, how do I deal with the guilt of now doing something that feels right for me instead of what’s obviously best for him?! Pumping is tiresome, I find it very hard to do other things like make healthy lunches for myself for example, and I’m going nuts with constantly washing and storing the paraphernalia.

—postpartum (4 months, as reported by mother)

Even though some mothers were themselves ready to stop pumping, they described difficulties related to family members who wanted them to continue to pump.

I am mentally and physically exhausted from the strenuous schedule of pumping and taking care of a newborn. I return to work in one week and don’t want to stress about pumping there. Not to mention physically I am fed up [with] having boobs that weigh a million pounds, sweat, and hurt when I don’t pump every 2 to 3 h, specifically if I try to sleep longer. [My husband] wants me to continue to pump [because] he feels like the benefits outweigh all the physical, mental and emotional stresses I’m going thru. What would You do?

—postpartum (1.5 months, as reported by mother)

Some mothers reported or anticipated physical discomfort (e.g., painful engorgement) that would result from stopping pumping and asked for advice on how to manage it. Both mothers who experienced and anticipated this discomfort asked how long it typically took for HM supply to dry up. Mothers who anticipated this discomfort in the future asked whether weaning gradually or all at once would lead to less pain.

## 4. Discussion

The findings presented here provide the first detailed insights into the information women seek from each other online about pumping and providing pumped HM. In this work, we identified a range of questions asked by pregnant and lactating women about these practices, which are paraphrased in Table 3. These questions suggest that mothers may benefit from additional guidelines as well as from both lay and professional support. The questions also suggest modifiable constraints to women’s practices for pumping and providing pumped HM at home and at work.

### 4.1. Guidelines for Safe Handling of Pumped HM

Our findings highlight the need for comprehensive, evidence-based best practice guidelines for safe storage and preparation of pumped HM. Previous research has shown that the additional handling involved in pumping, storing, and preparing pumped HM may increase the risks of infant infections [12,25]. In this study, mothers asked about a range of strategies for handling pumped HM, some of which may increase the risk of contamination of milk (e.g., mixing milk from multiple pumping sessions before storing it or preparing it to be fed to infants). These findings support the results from previous qualitative research [13], revealing that some pumped HM underwent a number of temperature and container changes before it was fed to infants. In combination, these findings and those of other researchers may partly explain why IFPS II infants who were bottle-fed HM exhibited more coughing and wheezing episodes in their first year than those fed at the breast [10].

### 4.2. Guidelines for Obtaining Pumps and Earlier Access to Pumps

There is also an indication of a need for clearer guidelines from insurance providers on how to obtain pumps as well as earlier access to pumps. In this study, we observed that many women relied on their health insurance policies to obtain pumps. However, women asked a range of questions about how and where they could obtain pumps, which suggests that they need guidelines on this process that are more understandable and readily available. Furthermore, the frequency of these questions in the prenatal period suggests that some women may prefer and benefit from earlier access to pumps from their insurance providers. Previous research suggests that, for mothers of very low-birth-weight infants, pumping in the first hour postpartum is important to mothers’ long-term HM output [26,27]. The questions asked by pregnant women in this sample suggest that early access to pumps may also be important to a wider group of mothers. Taken with previous qualitative work [13], the findings reported here underscore that timely access to adequate pumps may be important to mothers’ pumping success.

### 4.3. Additional Support from Family Members

Many of the mothers on BabyCenter reported feeling emotionally burdened by pumping in the early postpartum period, which suggests that mothers may benefit from more support from their partners and families. Social support, such as education about breast pumps from friends and relatives, is positively associated with longer durations of feeding HM [28]. Mothers’ reported reasons for considering stopping, such as feeling tired from balancing pumping, cleaning pump supplies, and taking care of their infants, point to ways in which their partners and families may offer support.

### 4.4. Additional Support from Employers and Coworkers

Mothers on BabyCenter also reported obstacles specific to pumping at work, which indicate that they may benefit from more support from employers and coworkers in managing and reducing such obstacles. These obstacles, such as inflexible work arrangements and lack of a designated place to pump, may explain, in part, why working IFPS II mothers who only pumped at work stopped feeding HM earlier than mothers who also, or only, fed their infants at the breast at work [1,19]. Thus, for mothers who cannot feed at the breast at work, support from employers and coworkers is important to their success in providing HM to their infants for as long as they would like.

### 4.5. Limitations

The qualitative work described here is not without its limitations. First, the sample of posts came from an open group of women whose demographic characteristics are unidentified, so the findings may not be generalizable to the larger population. For example, the analyses may not have captured the knowledge sought by women of lower income, who may not have access to the Internet or smartphones. Second, some posts on BabyCenter were randomly and irretrievably removed from forums for unspecified reasons and, as such, it is likely that some posts related to pumping and providing pumped HM were not collected. However, we gathered data every other day until the end of data collection period to reduce the number of missed posts. Third, when dividing posts into three intervals, we assumed that all infants in the April 2014 Birth Club were born on April 1, 2014. Thus, some questions may have been grouped in the incorrect postpartum interval, introducing unknown bias into the tally of questions in each interval. However, we expect such misclassification to be minimal in the prenatal and 0–1.5 months intervals, as many mothers indicated whether they were still pregnant or had recently delivered. Moreover, we do not anticipate that this limitation materially impacted the content or reliability of these findings. Finally, we did not analyze the responses to the women’s questions. Thus, the knowledge exchanged among mothers through their responses is an area of interest for further research. 

### 4.6. Strengths

This work also has several strengths. First, the study methods and analyses were informed by analyses of IFPS II data [23] and by findings from previous qualitative work [13]. This allowed us to group posts so that we could examine mothers’ practices of pumping and providing pumped HM both cross-sectionally and over time. Moreover, this allowed us to provide support to existing literature that has begun to identify potential determinants, practices, and consequences of pumping and/or providing pumped HM. Second, this study made use of mothers’ own words to understand their attitudes, perceptions, and practices related to pumping and providing HM. This minimized the question interpretation error commonly associated with other data collection methods, such as surveys. Third, this study was conducted after the implementation of the Affordable Care Act [29]. This allowed for documentation of mothers’ reactions and experiences with the new law, which was not possible with previous studies. This strength may result in a dataset that more closely reflects the experiences of current and soon-to-be mothers, who may now be more likely to obtain pumps through their insurance policies. Fourth, and most importantly, these findings identified common questions that mothers have about pumping and providing pumped HM. Women may benefit from having answers to these questions provided by their health professionals.

## 5. Conclusions

The findings presented here illustrate a wide range of information that women sought on social media about pumping and providing pumped HM to their infants. This information may represent what women are not receiving from their health professionals or insurance providers. This information may also suggest modifiable constraints to women’s practices for pumping and providing their milk. The questions women asked about how to safely store and prepare their pumped HM indicate their need for comprehensive, evidence-based guidelines for these practices. Furthermore, these findings highlight that mothers and their infants may benefit from early receipt of pumps and access to clearer guidelines from insurance providers on how to obtain pumps. Finally, this study highlights the difficulties women face in attempting to pump their milk at home and at work and, thus, identifies potential avenues through which families and employers may help women meet their goals for pumping and providing HM.

## Figures and Tables

**Table 1:** Number of posts by infant age and theme.

Main Theme	Infant Age (Months)			
	Prenatal	0–1.5 Months	1.5–4.5 Months	Total
**Choosing and purchasing pumps**	37	10	12	59
Storing and preparing pumped HM *	14	48	106	168
**Strategies for and difficulties with pumping**	12	118	103	233
**Integrating pumping into work**	5	10	37	52
**Stopping pumping**	0	4	27	31
**Total**	68	190	285	**543**

* HM = human milk.

**Table 2:** Themes and topics.

Main Theme	Examples of Topics Raised by Women
**Choosing and purchasing pumps**	Options available through insurance policy; length and logistics of insurance process; pump affordability vs. quality; when, where, and how to get a pump out-of-pocket
**Storing and preparing pumped HM**	Bag and bottle quality and desired traits (e.g., maintenance required to keep supplies clean and cost); length of storage time at room temperature, in refrigerator, and in freezer; thawing and warming; mixing from different pumping sessions; effects of alcohol, caffeine, and medications on pumped HM; smell, taste, and appearance of fresh, refrigerated, or frozen pumped HM
**Strategies for and difficulties with pumping and integrating pumping into work**	Pump features and malfunctions; managing physical discomfort; other experienced and anticipated barriers to pumping (e.g., heavy workload and a lack of designated place to pump)
**Stopping pumping**	Appropriate time to stop pumping and/or providing pumped HM; acceptable reasons to stop pumping; guilt; painful engorgement; drying up HM supply

**Table 3:** Themes and common questions.

Main Theme	Common Questions Asked by Women
**Choosing and purchasing pumps**	Which pumps are covered through my insurance policy, and how do I obtain a pump using my policy? When can I expect a pump obtained through my insurance policy to arrive? How do I acquire an affordable pump if I do not have an insurance policy that covers one? When should I purchase a pump out-of-pocket?
**Storing and preparing pumped HM**	Which bags/bottles should I purchase to store/provide pumped HM to my baby, and why? What containers should I use to store my pumped HM? How long can my pumped HM be stored at room temperature, in the fridge, and in the freezer before it is no longer safe for my baby to eat? Can I mix pumped HM from different pumping sessions before it is stored or fed to my baby? How should my pumped HM be thawed and/or warmed so that it is safe for my baby to eat? What do I need to do to avoid transferring a harmful amount of alcohol, caffeine, and medications to my baby through my milk? What should my HM taste/smell/look like when it is fresh and after it has been frozen and thawed, and how can I tell if it has gone bad?
**Strategies for and difficulties with pumping and integrating pumping into work**	What supplies do I need to use my pump effectively? What do I do if my pump stops working as well or at all? What do I do if I get nipple pain or bleeding after pumping? How do I pump enough milk when I have a heavy workload, a tough schedule, or inadequate places to pump?
**Stopping pumping**	When are other moms planning to stop pumping, and why? When and for what reasons is it okay to stop pumping and/or providing HM to my baby? How do I deal with the guilt I feel for deciding to stop pumping and providing pumped HM to my baby? How long does it take for my breast milk to dry up after I stop pumping? Should I stop pumping gradually or all at once?

This table lists questions that are not direct quotes, but rather questions that have been paraphrased by the authors to represent information commonly sought by women whose posts were analyzed.
